# The Vitamin D Receptor–BIM Axis Overcomes Cisplatin Resistance in Head and Neck Cancer

**DOI:** 10.3390/cancers14205131

**Published:** 2022-10-19

**Authors:** Aya Khamis, Désirée Gül, Madita Wandrey, Qiang Lu, Shirley K. Knauer, Christoph Reinhardt, Sebastian Strieth, Jan Hagemann, Roland H. Stauber

**Affiliations:** 1 Department of Otorhinolaryngology Head and Neck Surgery, Molecular and Cellular Oncology, University Medical Center, 55131 Mainz, Germany; 2 Oral Pathology Department, Faculty of Dentistry, Alexandria University, Alexandria 5372066, Egypt; 3 Centre for Medical Biotechnology (ZMB/CENIDE), Institute for Molecular Biology, University Duisburg-Essen, Universitätsstraße, 45117 Essen, Germany; 4 Center for Thrombosis and Hemostasis (CTH), University Medical Center Mainz, Langenbeckstrasse 1, 55131 Mainz, Germany; 5 Department of Otorhinolaryngology, University Medical Center Bonn, 53127 Bonn, Germany

**Keywords:** nuclear receptors, calcitriol, therapy resistance, platinum-based drugs, pro-apoptotic pathways, combination therapy, HPV

## Abstract

**Simple Summary:**

Treatment success of head and neck squamous cell carcinoma cancers (HNSCCs) is often hindered by cisplatin resistance. Vitamin D and its receptor (VDR) have been suggested to influence tumor pathobiology and therapy response. We found that VitD/analogs in combination with platinum-based drugs may help to fight therapy resistances in HNSCC. VitD/cisplatin combinations induced E-cadherin upregulation and killed cancer cells by increasing the expression of the pro-apoptotic protein BIM. By identifying the VDR/VitD/BIM axis, we here provide a molecular rationale for the anti-cancer activity of VitD/analogs in combination therapies, which should be further exploited in the clinics.

**Abstract:**

Treatment success of head and neck squamous cell carcinoma (HNSCC) is often hindered by cisplatin resistance. As inherent and acquired therapy resistance counteracts improvement in long-term survival, novel multi-targeting strategies triggering cancer cell apoptosis are urgently required. Here, we identify the vitamin D receptor (VDR) as being significantly overexpressed in tumors of HNSCC patients (n = 604; *p* = 0.0059), correlating with tumor differentiation (*p* = 0.0002), HPV status (*p* = 0.00026), and perineural invasion (*p* = 0.0087). The VDR, a member of the nuclear receptor superfamily, is activated by its ligand vitamin D (VitD) and analogs, triggering multiple cellular responses. As we found that the VDR was also upregulated in our cisplatin-resistant HNSCC models, we investigated its effect on overcoming cisplatin resistance. We discovered that VitD/cisplatin combinations synergistically killed even cisplatin-resistant cells at clinically achievable levels. Similar results were obtained for the clinically used VitD analog Maxacalcitol. Moreover, VitD/cisplatin combinations inhibited tumor cell migration by E-cadherin upregulation. Signaling pathway analyses revealed that VitD co-treatments triggered cancer cell death by increasing the expression of the pro-apoptotic BCL-2 family protein BIM. BIM’s pro-apoptotic activity in HNSCC cells was confirmed by ectopic overexpression studies. Importantly, BIM expression is positively associated with HNSCC patients’ (n = 539) prognosis, as high expression correlated with improved survival (*p* = 0.0111), improved therapy response (*p* = 0.0026), and remission (*p* = 0.004). Collectively, by identifying, for the first time, the VDR/BIM axis, we here provide a molecular rationale for the reported anti-cancer activity of VitD/analogs in combination therapies. Our data also suggest its exploitation as a potential strategy to overcome cisplatin resistance in HNSCC and other malignancies by inducing additional pro-apoptotic pathways.

## 1. Introduction

Head and neck cancers (HNCs) are among the top ten most common cancers, causing 5% of cancer death worldwide [1,2,3]. HNCs include tumors of the pharynx, the larynx, the lips, and the oral cavity, which predominantly emerge from mucous membranes. This so-called solid squamous cell carcinoma (HNSCC) represents the most common entity of HNC. Due to the aggressive heterogeneous nature of these tumors, anatomical inaccessibility, and high recurrence rates, survival rates for HNSCC have not improved significantly within recent decades [4,5]. Usually, treatment of HNSCCs includes surgery, radiation, and chemotherapy, or a combination of treatments [6]. First-line chemotherapy of HNSCCs predominantly includes cisplatin (cis-diamminedichloroplatinum II, CDDP)-based combination chemotherapy [1]. Unfortunately, cisplatin’s side effects and the development of chemoresistance have been limiting its effectiveness in clinics [1,6,7]. Despite the multiple attempts to overcome cisplatin resistance and minimize its dose, it is still the first and most effective line of treatment in many cases. Therefore, there is an urgent need to identify and establish novel combinations and/or alternative treatment options allowing the reduction in dose, and the development of resistances [1,8].

As shown by us and others, resistance to chemotherapeutics is indeed complex and not yet fully understood [1,8,9]. Overexpression of anti-apoptotic BCL-2 proteins and/or apoptosis inhibitors such as Survivin, in addition to transporters facilitating active influx and efflux of drugs, impact therapy resistance and overall survival in HNSCC [8,10,11]. Consequently, therapeutic strategies aim at shifting the cellular balance of anti-apoptotic in favor of pro-apoptotic proteins [10,11]. The intrinsic or mitochondrial pathway of programmed cell death plays an important role in killing cancer cells in response to various therapies and is regulated by reciprocal interaction between pro- and anti-apoptotic protein family members [10].

Dysfunction of nuclear receptor signaling contributes to a variety of proliferative diseases, including cancer, and drugs modulating nuclear receptor activity have been among the most potent agents in clinics for over 50 years [12]. Indeed, members of the nuclear receptor superfamily are key for almost all aspects of life, pathologies, and therapeutic intervention strategies [12,13,14]. Despite their long research history, their effects alone and/or in combination with other nuclear receptors have still not been resolved in detail. Moreover, various (patho)biological effects of nuclear receptors in the absence (‘ligand-independent’) and/or presence of their ligands (‘ligand-dependent’) have been reported [15,16]. The mechanistic details of these activities are not fully understood and thereby increase the complexity of nuclear receptor research and interpretation of observations [12,14,17]. Due to this complexity, we need to refer the reader to some comprehensive reviews for more details [12,14,17,18].

Among the members of the nuclear receptor superfamily, the vitamin D receptor (VDR) is among the most important members for which anti-tumoral effects have been already suggested [12,14,17,18]. Vitamin D (VitD) is mainly synthesized in the skin stimulated by ultraviolet-B (UV-B) exposure, as well as dietary intake. VitD is metabolized to 25-hydroxyvitamin D (25(OH)D3, calcifediol) in the liver, which is enzymatically catalyzed by CYP2R1. Further hydroxylation by CYP27B1 occurs in the kidneys to convert 25(OH)D3 into 1α,25- dihydroxyvitamin D3 (1,25(OH)2D3), which is also called calcitriol. Calcitriol exerts endocrine, autocrine, and paracrine effects as a steroid hormone and represents the active form of VitD binding to its receptor, the VDR [19,20]. Upon its binding to VDR, the receptor actively translocates to the nucleus, interacts with the retinoid X receptor (RXR), and initiates various transcription programs by binding to specific DNA elements (VDR-responsive elements = VDRREs) located in promoters and distal regions of target genes, such as CDKN1A, C-MYC, CDH1, and CYP24A1 [12,14,17,18,19,20,21]. Thereby, VDR, a member of the thyroid hormone-like NR subfamily, exerts various endocrine, autocrine, and paracrine effects [12,14,21]. VitD deficiency can result in various diseases, such as rickets in children, and osteoporosis [22], and seems to be (in)directly correlated with the occurrence of cancer [23,24]. Hence, previous studies reported that it reduces the risk of many cancer types, such as colon and breast cancer, and suggested VitD treatment for potential combination therapies [25,26,27]. However, although VitD treatment and VDR expression/activation have been suggested to influence various aspects of tumor pathobiology, disease progression, and therapy via numerous and sometimes controversial pathways [19], the mechanistic details remain to be dissected on the molecular level. In particular, little is known about VitD/VDR and its relevance for HNSCC and therapy resistances [28,29].

Consequently, we investigated the relevance of VitD/VDR in HNSCC patients and its potential role in cisplatin therapy resistance. By exploiting The Cancer Genome Atlas (TCGA) HNSCC data set, we demonstrate the additional clinical relevance of the VDR for HNSCC. Experimentally, we identified the role of the VDR and VitD as critical components for cisplatin resistance and evaluated the effects of the VitD/cisplatin treatment combinations on cell migration and apoptosis. Our study discovered that this drug combination efficiently eliminates even cisplatin-resistant HNSCC cancer cells, mainly by the induction of the pro-apoptotic protein BIM. Collectively, we recommend VitD/cisplatin treatments as a potential strategy to overcome cisplatin resistance in HNSCC and other malignancies.

## 2. Materials and Methods

### 2.1. Chemicals and Reagents

Unless stated otherwise, chemicals were purchased from Sigma Aldrich/Merck (Darmstadt/Munich, Germany) or MSC (MSC UG&CoKG, Mainz, Germany). Cell culture media and reagents were sourced from Gibco/Thermo Fisher Scientific (Dreieich, Germany). Disposables were purchased from Greiner Bio-One (Frickenhausen, Germany). The following antibodies were used (see also Supplementary Table S1): α-VDR (sc-13133; Santa Cruz Biotechnology, Heidelberg, Germany), α-VDR (ab3508, Abcam, Erlangen, Germany), α-GAPDH (sc-47724; Santa Cruz Biotechnology, Heidelberg, Germany), α-GFP (sc-8334; Santa Cruz Biotechnology, Heidelberg, Germany), α-Actin (A2066; Sigma Aldrich, Munich, Germany), anti-γH2AX (A300-081A, Bethyl Laboratories, US), α-BIM (B7929, Sigma Aldrich, Munich, Germany), and α-E-cadherin (610182; BD Biosciences, Heidelberg, Germany). Appropriate HRP-, Cy3-, or FITC-conjugated secondary antibodies (Sigma Aldrich, Munich, Germany; Santa Cruz Biotechnology, Heidelberg, Germany) were used. Reagents, such as cisplatin, were from Sigma (Sigma Aldrich, Munich, Germany) or MSC (MSC UG&CoKG, Mainz, Germany). 1α,25-dihydroxy vitamin D_3_ (calcitriol) was purchased from Sigma and Santa-Cruz (D1530, Sigma Aldrich, Munich, Germany and CAS 32222-06-3, Santa Cruz Biotechnology, Heidelberg, Germany).

### 2.2. Clinical Data Analysis

Publicly available gene expression and survival data sets were obtained from *The Cancer Genome Atlas* (TCGA), filtering for patients with HNSCCs (TCGA HNSC). Of note, expression values are not detectable for all genes of interest for every patient in the TCGA database. Here, whereas VDR expression was found in n = 612 patients, BIM expression was found in only n = 539 patients and analyzed as described [1]. Data were assessed via the USCS Xena server [30], and patients were grouped according to indicated phenotypic or clinical characteristics. Visualizations were performed with GraphPad Prism.

### 2.3. Cell Culture

Authenticated and characterized cell lines FaDu and SCC-4 were purchased from the *ATCC* repository and expanded, with stocks prepared at early passages and frozen stocks kept in liquid nitrogen. SCC-4 cells were established from a tongue squamous cell carcinoma. HNSCCUM-02T was established from tongue squamous cell carcinoma as described by Welkoborsky et al. [31]. The Pica cell line was established from laryngeal squamous cell carcinoma and maintained as described [1]. FaDu cell line was established from a hypopharyngeal squamous cell carcinoma [32]. Thawed cells were routinely monitored by visual inspection and growth curve analyses to keep track of cell-doubling times, and were used for a maximum of 20 passages for all experiments. Depending on the passage number from purchase, cell line authentication was further performed at reasonable intervals by short tandem repeat (STR) profiling. We cultured the HNSCCUM-02T and SCC-4 cells in Dulbecco’s Modified Eagle’s F-12 medium. Pica and FaDu cells were cultured in Dulbecco’s Modified Eagle’s Medium. We added 10% fetal bovine serum (FBS), and 1% penicillin–streptomycin to all medium types. Cells were cultured under a 5% CO_2_ atmosphere at 37 °C and subcultured every 3 days as described [1]. We checked the absence of mycoplasms regularly via the Venor GeM Advance detection kit (Minverva Biolabs, Berlin, Germany) according to the manufacturer’s instructions. Cell numbers were determined using Casy Cell Counter and Analyzer TT (OMNI Life Science GmbH & Co KG, Bremen, Germany). To treat the cells, Hy-clone fetal bovine serum (FBS) (Sigma Aldrich, Munich, Germany) was used instead of standard FBS to ensure the lack of vitamin D in the controls and control the treatment doses in the treated samples.

### 2.4. Generation of Cisplatin-Resistant Cell Model 

We generated constantly selected cell lines by treatment with doses of cisplatin corresponding to IC90 (5 µM) and then constant treatment (3 µM). We started the first experiments 6 months after constant exposure and re-establishment of regular proliferation.

### 2.5. Cell Viability Assays

To probe cell viability, we seeded the cells in 96-well plates (5000 to 10,000 cells/well) according to the cell line and the treatment period and treated them with indicated substances and concentrations (n = 3) starting 24 h after seeding. Then, 48/72 h after treatment, we performed a commercial assay CellTiter-Glo^®^ 2.0 (Promega, Walldorf, Germany) according to the manufacturer’s instructions and recorded luminescent signals using a Tecan Spark^®^ (Tecan Group Ltd., Männedorf, Switzerland). Signals were normalized to untreated control samples. 

### 2.6. Fluorescence Microscopy

Fluorescence images were acquired, analyzed, and quantified using Axiovert 200 M fluorescence microscope (Zeiss) or the automated high-content screening microscope Array Scan VTI (Thermo Fisher) as described [1,33,34]. We seeded cells in microscopic dishes (35 mm, MatTek) or clear-bottom 96-well plates (Greiner) and fixed them with 4% PFA (20 min, RT). For immunofluorescence staining, we additionally permeabilized the cells via incubation with Triton-X 100 (0.1%, 10 min, RT). Antibodies were diluted in 10% FBS/PBS and incubated with samples for 1 h at RT. We washed the cells (n = 3) in (PBS) and then incubated the samples with fluorophore-labeled antibodies for 1 h at RT. Finally, we stained the nuclei by adding Hoechst 33342 (50 ng/mL in PBS) for 30 min at RT. For automated high-content screening, regions of interest were created using the nucleus signal and each sample was acquired in triplicate, imaging at least 5000 events per sample according to [1].

### 2.7. Cell Migration Assay/Wound-Healing Assays

We seeded a monolayer of cells in 6-well dishes. After 24 h (cells 80–90% confluent), we created a wound by scratching the surface with a plastic 200 μL pipette tip. Then, we changed the medium with the corresponding previously mentioned treatments. The cells were cultured under the experimental condition medium mentioned above with cisplatin in the presence or absence of vitamin D at 37 °C over 72 or 96 h. The wound size was documented periodically every 24 h over 72 or 96 h via microscopy. We assessed and calculated the wound size using ImageJ software. The relative wound size was determined and visualized using GraphPad Prism.

### 2.8. Plasmids and Transfection

To construct a VDR expression plasmid, cDNA was isolated from HNSCC cancer cell lines, and the full open reading frame of human VDR cDNA was cloned into the pcDNA3.1 mammalian expression vector (Invitrogen) with C-terminal GFP-tag (for primer sequences, please see Supplementary Table S2). Colony PCR was performed to check for positive clones [33,34,35]. The expression construct for human BIM_EL_ pCDNA4/TO-BIMEL was described before [36]. For expression of a BIM_EL_-GFP fusion, BIM_EL_ cDNA was PCR-amplified and cloned into pc3-GFP (pc3BIM_EL_-GFP) as stated before [30].

For cellular transfection, plasmid DNA and Lipofectamine 3000 (Fisher Scientific, Schwerte, Germany) were mixed according to the manufacturer’s instructions and added to the cells, which were cultured in an Opti-MEM medium as described [37].To mark VDR-expressing cells, plasmid pC3 coding for GFP expression was co-transfected. To exclude artifacts, a control transfection of empty plasmid pC-DNA3 and the GFP-coding plasmid was conducted in parallel. The medium was changed 5 h post-transfections to a normal cell culture medium. We confirmed VDR overexpression of cell lines via Western blot analysis, and positive transfectants were selected by addition of puromycin (1 µg/mL; Sigma Aldrich, Munich, Germany). To establish a uniform expression of the VDR-transfected cells, the cells were sorted into low, medium, and high fluorescence using FACS as described before [35].

### 2.9. Protein Extraction, Immunoblot Analysis 

Whole-cell lysates were prepared using low-salt lysis RIPA buffer (50 mM Tris pH 8.0, 150 mM NaCl, 5 mM EDTA, 0.5%NP-40, 1 mM DTT, 1 mM PMSF, Complete EDTA-free from Roche Diagnostics, Mannheim, Germany), and samples were separated on 12% SDS gels as has been described before [35,38,39]. Blotting onto activated PVDF membranes was achieved with a Trans-Blot Turbo (Bio-Rad, Feldkirchen, Germany), and blocking as well as antibody incubations (1 h/RT or 16 h/4 °C depending on the antibody) were performed in 5% milk powder or BSA in TBST or PBS. Detection of the luminescence signal of HRP-coupled secondary antibodies after addition of Clarity Western ECL Substrate was performed using the ChemiDoc^TM^ imaging system (Bio-Rad). Equal loading of lysates was controlled by reprobing blots for housekeeping genes Actin (Munich, Germany) or GAPDH (Heidelberg, Germany). At least n = 2 biological replicates were performed, and representative results are shown. Results of densitometric analyses of all Western blots can be found in the Supplementary Materials.

### 2.10. Statistical Analysis

Statistical analyses were performed using GraphPad Prism (version 9.3.1) as described [1]. Survival data were obtained from the USCS Xena server, visualized, and analyzed by GraphPad prism (Log-rank/Mantel–Cox test; hazard ratio (Mantel–Haenszel)). For two groups, a paired or unpaired Student’s *t*-test was carried out, and for more groups, analysis of variance (ANOVA) was performed. Unless stated otherwise, *p* values represent data obtained from two independent experiments carried out in triplicate. Statistical significance is represented in figures as follows: * *p* < 0.05, ** *p* < 0.01, *** *p* < 0.001, and **** *p* < 0.0001, and n.s. indicates not significant. A *p* value that was less than 0.05 was considered statistically significant.

## 3. Results

### 3.1. Clinical Relevance of Vitamin D Receptor (VDR) in HNSCC Patients

**Figure 1:**
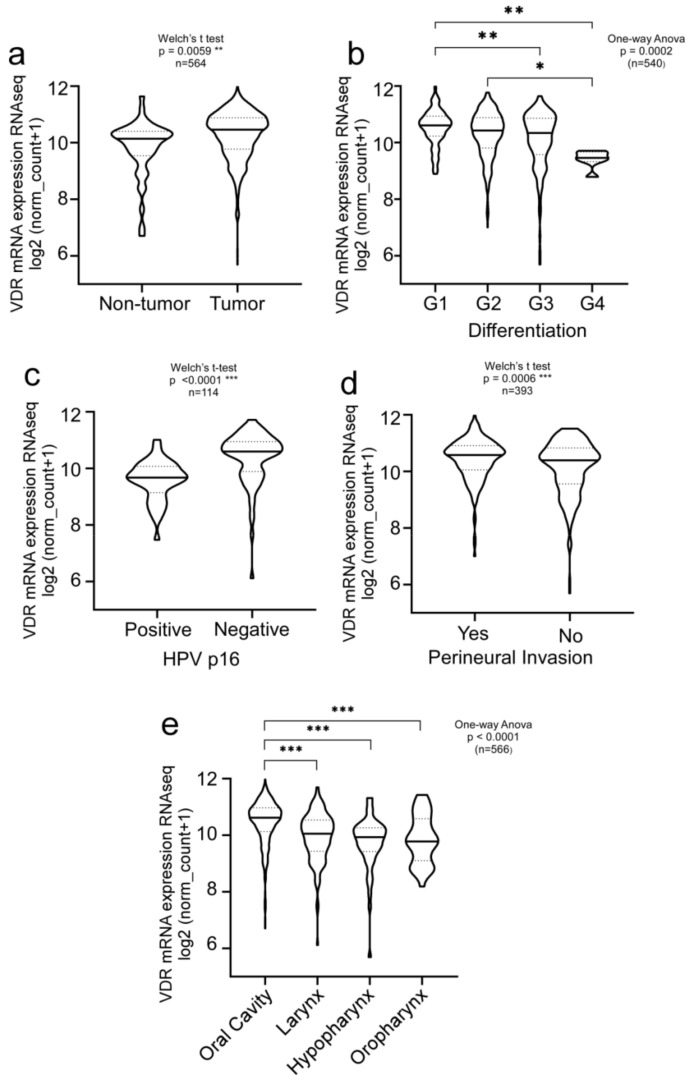
Clinical relevance of VDR in HNSCC patients. VDR is significantly overexpressed in well-differentiated, invasive HNSCCs. Bioinformatic analysis of the TCGA HNSCC cohort (n = 604). Overexpression of VDR was found in (**a**) primary tumors versus normal tissue and correlates with (**b**) tumor differentiation, (**c**) HPV status, (**d**) perineural invasion, and (**e**) tumor localization. Significant *p* values and sample size (n) are indicated. *, *p* < 0.05; **, *p* < 0.01, ***, *p* < 0.005.

We first investigated VDR expression patterns and their correlation with clinical parameters in the PANCAN data set obtained from *The Cancer Genome Atlas* (TCGA), comprising more than 12,000 samples of cancer patients of various entities and clinical backgrounds. Comparing different malignancies, high expression of the VDR was found not only in colon adenocarcinomas but also in HNSCC, supporting a potential pathobiological relevance of the VDR for this tumor entity (Supplementary Figure S1). We not only found the VDR to be significantly overexpressed (n = 564, ** *p* = 0.0059) in tumors but also that high VDR expression correlated with the histological differentiation of the tumor (Figure 1a,b). Since the HPV status affects the therapy outcome and prognosis of HNSCC patients, we also compared HPV-negative versus HPV-positive patients. VDR expression was significantly increased in HPV-negative HNSCC patients (Figure 1c; n = 114; *p* < 0.0001). 

Moreover, high VDR expression correlated with perineural invasion (Figure 1d, n = 393; ** *p* = 0.0006), indicating that VDR upregulation may stimulate the capability of tumor cells to metastasize. Moreover, VDR expression correlated with HNSCC tumor localization, such as the oral cavity, larynx, oro-, or hypopharynx (Figure 1e, n = 566, *p* < 0.0001).

### 3.2. VDR Overexpression Contributes to Cisplatin Resistance

**Figure 2:**
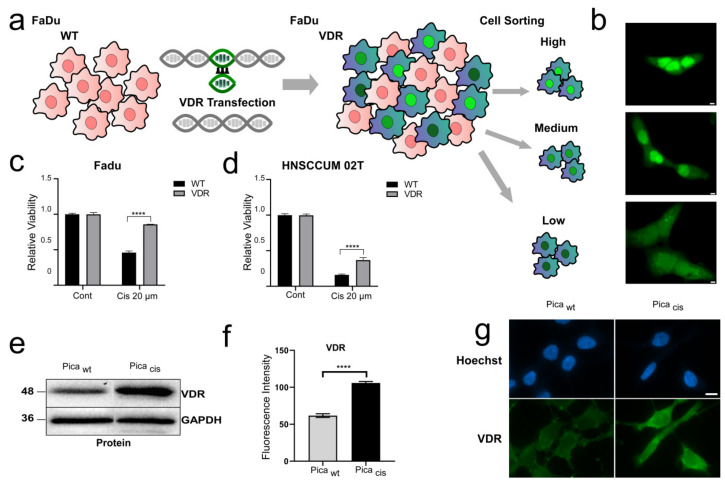
VDR overexpression contributes to cisplatin resistance. (**a**) Generation of VDR-overexpressing HNSCC cell models. Fadu cells were transfected with VDR C-terminally fused to GFP (green). Positive clones were selected using puromycin, and sorted by FACS into high, medium, and low subpopulations. Low-expressing subpopulations were used for experiments. (**b**) Fluorescence-microscopy-visualized VDR expression levels in stable HNSCC (Fadu) cell line. Scale bar, 10 µm. (**c**,**d**) Viability assay revealed that VDR-overexpressing Fadu cells (**c**) and HNSCCUM-02T cells (**d**) are more resistant to cisplatin treatment. Cells were treated with 20 µM cisplatin for 72 h, and viability was measured and normalized to untreated controls (Cont). **** *p* < 0.0001. (**e**) Overexpression of VDR in resistant Pica_CIS_ versus sensitive (Pica_WT_) cells was demonstrated by immunoblot analysis. GAPDH served as loading control. (**f**,**g**) Immunofluorescent staining confirmed overexpression of endogenous VDR protein in cisplatin-resistant Pica cells (Pica_cis_). VDR expression was automatically quantified using the high-content screening platform Array Scan VTI. **** *p* < 0.0001. (**g**) Fluorescence-microscopy-visualized VDR overexpression in Pica_cis_ cells. Cells were stained with specific fluorescent VDR Ab (green), and nuclei marked with Hoechst (blue). Scale bar, 10 µm.

To experimentally investigate the impact of the VDR on cisplatin resistance in HNSCC, we established HNSCC cell lines stably overexpressing VDR fused to GFP. First, we cloned the VDR reading frame from primary HNSCC tumors, and stably transfected the VDR-GFP expression plasmid into HNSCC FaDu and HNSCCUM-02T cells (Figure 2a, Supplementary Figures S2 and S3). Fluorescence microscopic analyses revealed different expression levels in high, medium, and low subpopulations sorted by FACS (Figure 2b, Supplementary Figure S3). As we did not detect differences in the viability of endogenous versus low- or high-VDR-overexpression cell models, and to mimic in vivo conditions, we mainly used endogenous or low-ectopic-VDR-expression models for further characterization. Next, to analyze the effect of VDR overexpression on treatment response, we measured cell viability after treating cells with 20 µM cisplatin (Figure 2c,d). Cisplatin treatment resulted in decreased viability of all tested cells. Interestingly, VDR-overexpressing FaDu and HNSCCUM-02T cells were more resistant to cisplatin treatment compared to wild-type (wt) cells, further indicating a role of VDR expression in chemoresistance.

To further verify our hypothesis, we additionally established a cisplatin-resistant HNSCC cell line, allowing the comparison of cisplatin-sensitive versus -resistant cells (Pica_WT_/Pica_Cis_). Here, Western blot analysis (Figure 2e) and immunofluorescence staining (Figure 2f,g) demonstrated overexpression of the VDR in the resistant cell line. Moreover, VDR overexpression could be objectively quantified using our automated high-throughput microscopy platform (Figure 2f). To underline the relevance of our findings using an additional independent cell model, we established a second cisplatin-resistant cell line, Fadu_Cis_ (Supplementary Figure S4). Similar to the results obtained in the Pica cell model, immunoblot analysis confirmed increased VDR expression in the cisplatin-resistant Fadu_Cis_ cells (Supplementary Figure S4). 

### 3.3. Ligand-Dependent VDR Expression/Activation Overcomes Cisplatin Resistance

**Figure 3:**
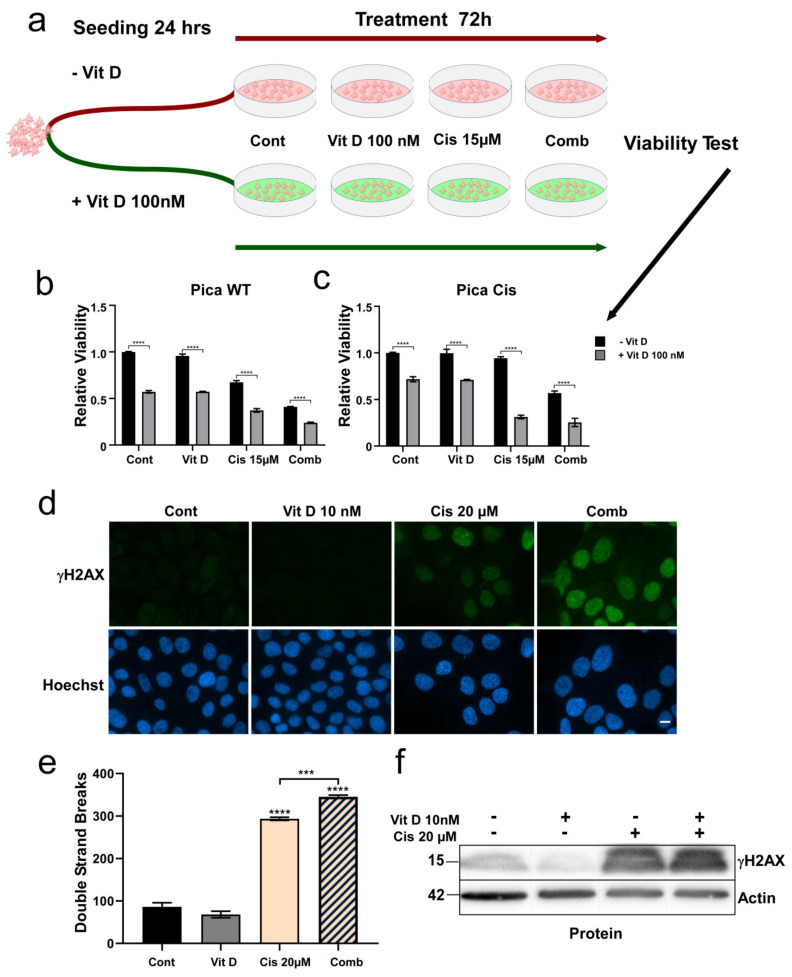
VitD co-treatment allows HNSCC to overcome cisplatin resistance. (**a**) Scheme illustrating the VitD pre-treatment schedule, which mimics physiological high versus low VitD serum levels. Cells were seeded in the presence or absence of 100 nM VitD; after 24 h, cells were treated with 20 µM cisplatin with and without VitD. (**b**,**c**) Combinational treatment of VitD/cisplatin synergistically triggered cell death of not only the cisplatin-sensitive (**b**) but also the highly resistant Pica cis cells (**c**). Cells were treated as described in (**a**). The viability of the cells was measured and normalized to untreated controls. (**d**) Fluorescence microscopy was used to visualize DNA damage. Compared to the VitD/cisplatin combination treatment, even cisplatin-treated cells showed a lower number of DNA damage events (quantified as γH_2_AX damage foci/per cell). Cells were treated for 24 h (10 µM VitD; 20 µM cisplatin), and γH_2_AX foci were detected by specific antibodies. Scale bar, 10 µm. (**e**) DNA damage events were automatically quantified by high-throughput microscopy and normalized to untreated controls. ***, *p* < 0.005, **** *p* < 0.0001 (**f**) Immunoblot analysis confers significantly increased γH_2_AX levels in VitD/cisplatin co-treated HNSCCUM-02T cells. Actin served as the loading control.

Our clinical and experimental data support a role of the VDR in HNSCC and cisplatin resistance, which is in line with the ‘ligand-independent’ effects described for various pathologies [16,40,41]. We next tested if ‘ligand-dependent’ VDR activation by VitD/analogs in combination with chemotherapeutic stressors, such as cisplatin, may even trigger cancer cell death of cisplatin-resistant cells. Here, we probed various HNSCC cell lines, including our cisplatin-resistant models, and measured cell viability after VitD/cisplatin combination treatment schedules. When referring to vitamin D, we used the active form calcitriol (1,25(OH)_2_D_3_), if not indicated otherwise. To mimic physiological conditions of high and low VitD serum levels, cells were seeded in the presence or absence of 100 nM Vitamin D (Figure 3a). After 24 h, cells were additionally treated with physiological concentrations of VitD (10–100 nM) or 15µM cisplatin alone, and with the combination. Corresponding dose–response curves and IC50 values are shown in Supplementary Figure S5. As expected, VitD alone did not affect cell viability. In contrast, combinational treatment of VitD/cisplatin synergistically triggered the cell death of not only the cisplatin-sensitive cells but, importantly, also the highly resistant cells (Figure 3b,c). The synergistic effect of the VitD/cisplatin combination on cell vitality was independently confirmed for three other HNSCC cell lines (Supplementary Figure S6). Importantly, similar results were obtained for the clinically approved VitD analog Maxacalcitol (Supplementary Figure S7), for which fewer side effects, such as phosphatemic properties, have been reported [42,43,44].

VitD-dependent sensitization of cancer cells to cisplatin-induced genotoxicity was independently verified by a DNA damage assay quantifying γH_2_AX damage foci (Figure 3d–f). Here, cisplatin-induced DNA damage events were significantly increased by VitD co-treatment.

### 3.4. VitD Treatment Reduces Migration of HNSCC Cells

**Figure 4:**
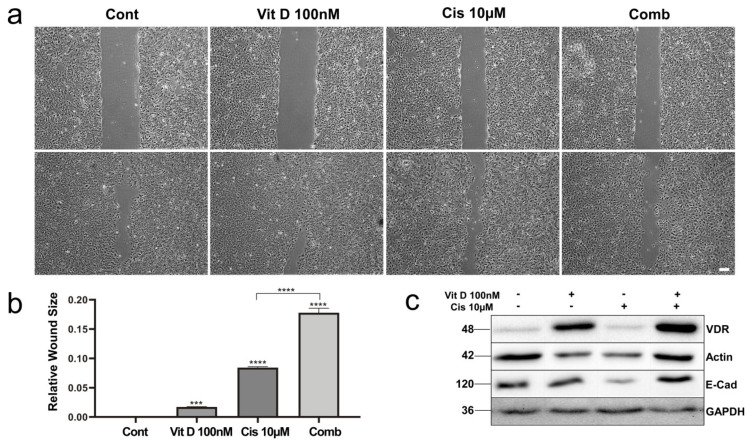
VitD reduces the migration ability of HNSCC cells. (**a**) Wound-healing assays were performed to evaluate the migration of HNSCCUM-02T cells. Cells were treated with VitD (100 nM) or cisplatin (10µM) alone, and with the combination. Wound size is shown for 0 h (upper panel), and after 72 h (lower panel). Scale bar, 100µm. (**b**) Relative wound size after 72 h was significantly increased after combination treatment of VitD/cisplatin. ***, *p* < 0.005, **** *p* < 0.0001. (**c**) Immunoblot analysis shows upregulation of VDR and E-cadherin in response to VitD, counteracting the cisplatin-induced downregulation of E-cadherin. Actin and GAPDH served as the loading control. Proteins were detected by specific Ab.

As our clinical data also revealed that high VDR expression correlated with perineural invasion (Figure 1d), indicating that VDR expression may stimulate the capability of tumor cells to migrate. We investigated the impact of VitD on the motility of HNSCC cells. Wound-healing assays demonstrated that VitD treatment inhibited the migration of HNSCCUM-02T (Figure 4) and Pica cells (Supplementary Figures S8 and S9). Again, co-treatment of VitD together with cisplatin further increased the drug sensitivity of the cells, resulting in further decreased mobility (Figure 4a,b). Molecularly, we found that VitD treatment not only enhanced VDR expression but also restored E-cadherin levels, which were decreased upon cisplatin treatment (Figure 4c, Supplementary Figure S9c). As E-cadherin counteracts EMT, which is required for cell migration and metastasis, these results suggest that the effects of VitD on tumor cell migration depend (at least) on the upregulation of E-cadherin. 

### 3.5. The VitD/VDR/BIM Axis Aids in Overcoming Cisplatin Resistance in Head and Neck Cancer

**Figure 5:**
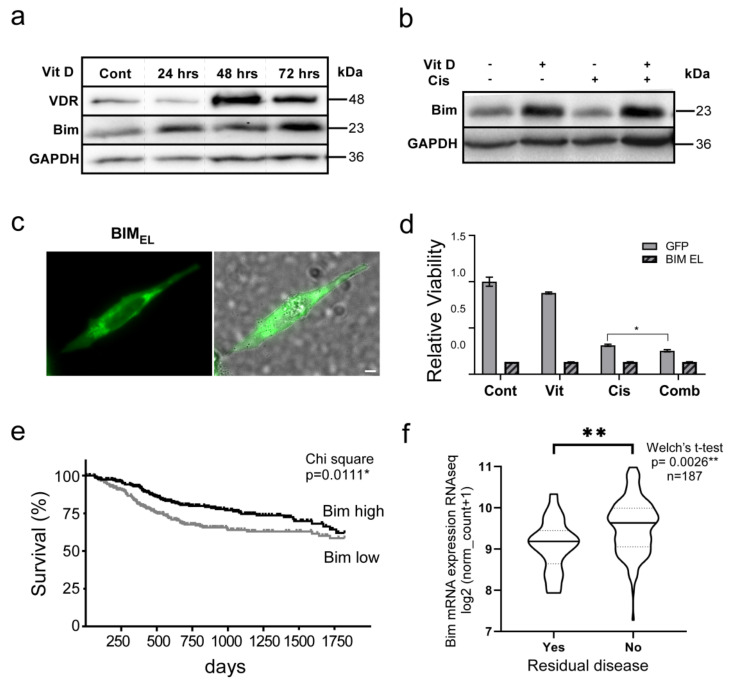
The VitD–BIM axis aids in overcoming cisplatin resistance in head and neck cancer. (**a**,**b**) Immunoblot analyses reveal significant increase in VDR and BIM in VitD and VitD/cisplatin co-treated HNSCCUM-02T cells. GAPDH served as the loading control. (**c**) Fluorescence microscopy was used to visualize cytoplasmic BIM-GFP (BIM_EL_/extra-long isoform) expression in Pica cells. Cells were analyzed 16 h post-transfection. Scale bar, 10 µm. (**d**) Ectopic BIM-GFP expression triggers apoptosis of HNSCC cells. GFP was used as control. Cells were treated for 72 h (100 µM VitD; 20 µM cisplatin, and combination). Cell viability was normalized to untreated controls. (**e**) Survival analysis demonstrates that high BIM expression correlates with improved disease-specific survival of HNSCC patients. n = 539; *p* = 0.0111*. Hazard ratio (Mantel–Haenszel) = 1.528. BIM low < 9.458 (median), and BIM high ≥ 9.458. (**f**) High BIM expression significantly correlates with improved therapy success, as shown by the status after primary therapy (disease after curative treatment). *p* values and sample size (n) are indicated. *, *p* < 0.05; **, *p* < 0.01.

To dissect the molecular mechanisms of how VitD co-treatment executes its pro-apoptotic effects, we analyzed the expression of various pro- and anti-apoptotic proteins in VitD/cisplatin-treated HNSCC cells. Interestingly, we found that VitD treatment-induced expression of VDR was accompanied by increased expression of the pro-apoptotic BH3 domain-only protein BIM in HNSCCUM-02T (Figure 5a), Pica (Supplementary Figure S9), and Fadu cells (Supplementary Figure S10). Importantly, VitD/cisplatin co-treatment was most potent in upregulating Bim (Figure 5b), providing a molecular explanation for the efficient cancer cell-killing activity we discovered, even for cisplatin-resistant cells. Of note, similar results were obtained for Maxacalcitol (Supplementary Figure S7) 

To unambiguously verify that enhancing BIM levels indeed triggers apoptosis of HNSCC cells, we first cloned BIM expression plasmids from primary HNSCC tumors. Ectopic overexpression studies of plasmids encoding a BIM_EL_-GFP fusion or untagged BIM_EL_, the longest BIM isoform (196 amino acids) resulted in efficient cell death of all cell lines tested (Figure 5c,d, Supplementary Figure S10, and not shown). These results provide strong evidence that BIM is critical for the observed VitD/cisplatin-induced killing of HNSCC cells.

We further examined the potential clinical relevance of BIM by analyzing the TCGA HNSCC data set. Here, high BIM expression indeed correlated with improved disease-specific survival of HNSCC patients (n = 539, *p* = 0.0111) (Figure 5e). As shown in Figure 5e, HNSCC patients with low BIM levels had a 52.8% increased risk of death (hazard ratio = 1.528). BIM’s relevance is further supported by markers of therapy success, such as residual disease after first-line treatment (Figure 5f) and primary therapy outcome (Supplementary Figure S11). Here, patients who exhibited no residual disease and showed complete tumor remission expressed significantly higher amounts of BIM compared to patients who underwent additional treatments with progressive disease. Collectively, our experimental and clinical data underline the relevance of VitD/VDR cisplatin-induced BIM expression for HNSCC.

## 4. Discussion

As treatment of HNSCC and most cancers is often complicated by therapy resistance, strategies triggering cancer cell apoptosis as well as the mechanistic identification of the underlying mechanisms are needed. Various clinical and laboratory studies investigated the influence of VitD and its receptor VDR on cancer incidence, prognosis, aggressiveness, and therapy. However, the mechanistic details are not always resolved and various conflicting reports underline the complexity of the VDR’s role in (patho)biologies [45]. Our study here revealed that the VDR is upregulated in HNSCC, and VDR expression correlated with clinically relevant phenotypes. Taken together, our analyses of clinical data suggest the VDR as a marker of invasive HNSCC tumors with the potential to metastasize. We can only speculate on the correlation between VDR levels and the HPV status of tumors. Similar to various observations, the molecular details of HPV infections and cancer hallmarks await dissection. Additionally, the clinical significance of VDR overexpression is controversially discussed for different cancer entities, suggesting both tumor-promoting and tumor-suppressing activities [30,46,47]. In line with our findings, there are studies correlating VDR overexpression with negative prognostic factors, such as the occurrence of metastases and reduced (progression-free) survival [46]. On the other hand, high VDR expression has been associated with improved prognosis of patients with lung [48], prostate [30], pancreatic [49], colorectal [47], and bladder cancers [50].

It has to be mentioned that the analyzed TCGA HNSCC data set does not contain information about the VitD serum levels of the patients. As shown in our study, exposure to its ligand VitD leads to induction of VDR expression on a cellular level. Thus, differential VitD levels at the time of tumor development/diagnosis can lead to a bias in the analyzed data. These challenges in the analysis of VitD-associated studies have been discussed before by Pilz et al., who suggest that significant differences regarding the individual molecular responses to VitD (due to the personal VDR status) have to be considered [27]. In the future, further prospective studies are needed taking into account not only the expression of the VDR but also VitD serum levels of the analyzed HNSCC patients. For example, the study of Bochen et al. could identify low VitD levels as a significant predictor of poor overall survival [51]. Thus, stratification of patient cohorts according to combined VitD level–VDR expression profiles will certainly reveal a more realistic picture of how these variables indeed influence the prognosis and survival of HNSCC and cancer patients in general.

Here, we could show that experimentally induced (by stably expressing VDR-GFP), as well as naturally occurring upregulation of the VDR (in cisplatin-resistant cell lines) was accompanied by increased resistance towards cisplatin treatment. This is in line with previous studies showing that overexpression of the VDR appears in chemoresistant colon cancer cells [52]. However, to the best of our knowledge, there is no study for head and neck cancers so far showing that overexpression of the VDR correlates with enhanced cisplatin resistance. Remarkably, chemoresistance of the cells was acquired in the absence of the natural VDR ligand, vitamin D. Here, ligand-independent activation of the VDR might play a role in promoting cisplatin resistance. It has been reported before that the VDR can execute different cellular functions in a ligand-dependent and also -independent manner [16,53]. Interestingly, prolactin expression seems to contribute to chemoresistance in breast and ovarian cancers [54,55]. However, the detailed mechanism of such ligand-independent VDR-mediated gene activation in HNSCC and cancer cell in general, and how it might be involved in the development of resistances, must be dissected in further studies. 

Our results now suggest that VDR/VitD plays a major role in tumor pathogenesis, progression, and therapy response for HNSCC as well. In addition to the ligand-independent effects of VDR overexpression we discussed before, our study also revealed that VitD-mediated activation of VDR overcomes cisplatin resistance in HNSCC cells. In all tested cell models, the presence of VitD sensitizes (chemoresistant) cancer cells to cisplatin-induced cell death. A VDR-mediated enhanced response to cisplatin treatment by VitD has been also suggested for other cancer types, potentially involving the NFκB pathway [29,50].

As shown in our study, combined VitD/cisplatin treatment was not only accompanied by increased cell death and DNA damage, but also reduced the migration potential of HNSCC cells. Here, by restoring high E-cadherin levels, and thus counteracting epithelial mesenchymal transition (EMT), which is key for metastasis, VitD exhibits the potential to reduce the metastatic potential of tumor cells. A recent study also suggested the involvement of VitD in the metastasis of mammary cancer via E-cadherin expression [56], supporting our findings. Moreover, results of large clinical trials suggest that VitD supplementation may reduce the incidence of advanced (metastatic or fatal) cancers [51,57]. Taken together, these results suggest a role of VitD/VDR in HNSCC metastasis as well, and that VitD treatments may prevent cancer from spreading (at least) by affecting E-cadherin-regulated EMT.

Cisplatin resistance and advanced HNSCC result mostly from ineffective attempts of the tumor cells to undergo apoptosis [6]. With the identification of the VitD/VDR/BIM axis, we suggest a potential strategy that may help to overcome cisplatin resistance in HNSCCs. Clearly, as shown here in tumor cell lines and patients, BIM is an important clinically relevant HNSCC disease mediator. Due to the multi-pathway effects of VitD, it is challenging to dissect a single mechanism responsible for cancer-associated VitD effects. We are aware that VitD/VDR has been suggested to affect various cancer-relevant signaling pathways, such as Akt- and/or mTOR [58,59], which we did not analyze in detail here. This might be considered a limitation of our and many other studies on VitD/VDR. Hence, although our data support a major role of the identified VitD/VDR/BIM axis for HNSCC pathologies, other pathways and chemo-genetic modulators deserve future investigations. Additionally, we did not investigate the impact of VitD/VDR on other chemo-radiation therapeutic strategies, such as antibodies or kinase inhibitors. However, we feel that our report will stimulate the field to examine the benefits of such additional VitD combinations in future studies.

## 5. Conclusions

**Figure 6:**
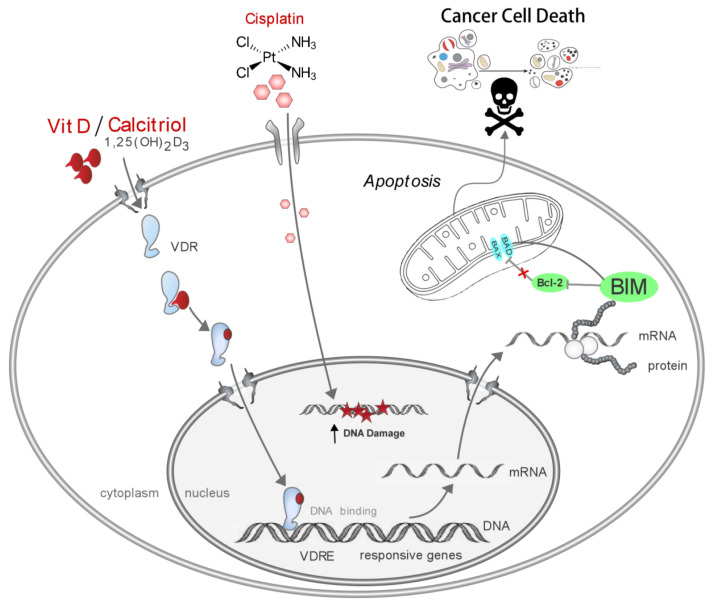
Model summarizing how the VitD/VDR/BIM axis overcomes cisplatin resistance in head and neck cancer. VitD treatment is able to sensitize HNSCC cells to cisplatin-induced cell death via induction of DNA damage and activation of pro-apoptotic pathways. After cellular uptake, VitD binds to VDR, which then translocates to the nucleus and binds to specific promoter sites, thereby inducing multiple pathobiological pathways. In HNSCC, the VitD/VDR complex also induces the expression of the pro-apoptotic protein BIM. BIM efficiently induces the activation of mitochondria-dependent apoptosis, and together with cisplatin-induced DNA-damage, efficiently triggers cancer cell death, including that of cisplatin-resistant cancer cells. Simplified schematic illustration of pathways and players, not drawn to scale.

Following a tiered pipeline from clinical hypothesis formation over bioinformatic analysis to in vitro confirmation, we conclude that VitD/analogs in combination with platinum-based drugs may help to fight therapy resistances in HNSCC and other malignancies. Our conclusion is based on the following findings: First, VDR is overexpressed in tumors of HNSCC patients, correlating with tumor differentiation, HPV status, and perineural invasion. Second, VDR’s relevance for cisplatin resistance was confirmed by ectopic expression studies and cisplatin-resistant cell lines. Third, VitD/cisplatin combinations synergistically killed even cisplatin-resistant cells and inhibited tumor cell migration. Mechanistically, VitD/cisplatin combinations induced E-cadherin upregulation and killed cancer cells by increasing the expression of BIM (Figure 6). Moreover, BIM induced apoptosis in HNSCC models and is positively associated with HNSCC patients’ therapy response and prognosis. Collectively, by identifying the VDR/VitD/BIM axis, we here provide a molecular rationale for the anti-cancer activity of VitD/analogs in combination therapies, which should be further exploited in clinics.

## 6. Recommendations

Our report, together with other studies, strongly suggests that VitD (co-)treatments should be exploited to overcome cisplatin resistance in head and neck, oral, and potentially other cancers. Although Figure 6 summarizes our findings, it should not be understood as a clinically ready ‘workable therapy proposal’. In this regard, many additional clinically relevant factors need to be considered. For example, we recommend that BIM expression levels in tumors also be monitored and that patients have their serum 25 hydroxyvitamin D (25(OH)D) level tested. Depending on the values, appropriate supplements could be given to increase the 25(OH)D serum levels to 70–80 ng/mL. However, such therapies need to be monitored carefully, including the appropriate clinical care. The results of such clinical studies need to be analyzed to better define recommended doses and treatment frequencies.

## Data Availability

The cell line raw data required to reproduce these findings are available upon request. The clinical results shown here are based on data generated by the TCGA Research Network: https://www.cancer.gov/tcga, accessed on 1 March 2022.

## Supplementary Materials

The following are available online at https://www.mdpi.com/article/10.3390/cancers14205131/s1: Table S1: Antibodies used for Western Blot and immunofluorescence analyses. Table S2: Primer Sequences. Figure S1: VDR mRNA expression in different tumor types according to the PANCAN data set obtained from The Cancer Genome Atlas (TCGA). Figure S2: Overexpression of VDR increases cisplatin resistance of FaDu cells. Figure S3: Overexpression of VDR increases cisplatin resistance of HNSCCUM-02T cells. Figure S4: VDR is upregulated in cisplatin-resistant HNSCC cell models. Figure S5: Dose-response curves and IC50 values for HNSCCUM-02T (a–c), FaDu (d–f), SCC-4 (g–i), and Pica cells (j–h). Figure S6: VitD treatment increases cisplatin sensitivity of different HNSCC cell lines. Figure S7: The VitD analog Maxacalcitol executes similar function on HNSCC cells in overcoming cisplatin resistance. Figure S8: VitD reduces the migration ability of HNSCC cells. Figure S9: Immunoblot analysis of Pica cells shows upregulation of VDR (a), BIM (b), and E-cadherin (c) in response to VitD/cisplatin. Figure S10: The VitD-BIM axis aids in overcoming cisplatin resistance in Fadu cells. Figure S11: High BIM expression significantly correlates with improved therapy success shown by primary therapy outcomes. Raw data file (Western blot raw data.pdf).
