# Supplementary material for: The Vitamin D Receptor–BIM Axis Overcomes Cisplatin Resistance in Head and Neck Cancer
Source: Cancers (Basel). 2022 Oct 19;14(20):5131. doi: 10.3390/cancers14205131 (PMC9600548; doi:10.3390/cancers14205131)
Supplement: Supplementary file 1 [file cancers-14-05131-s001.zip › cancers-1910430_Supplementary 2nd final.pdf]

cancers

MDPI

Supplementary Information

The Vitamin D receptor-BIM axis overcomes cisplatin resistance in head and neck cancer

Aya Khamis[1,2,*], Désirée Gül[1,*], Madita Wandrey[1], Qiang Lu[1], Shirley K. Knauer[3], Christoph Reinhardt[4], Sebastian Strieth[5], Jan Hagemann[1], and Roland H. Stauber[1,#]

1. Department of Otorhinolaryngology Head and Neck Surgery; Molecular and Cellular Oncology; University Medical Center, Mainz, Germany
2. Oral Pathology Department, Faculty of Dentistry, Alexandria University, Alexandria, Egypt
3. Institute for Molecular Biology, Centre for Medical Biotechnology (ZMB/CENIDE), University Duisburg-Essen, Universitätsstraße, 45117 Essen, Germany
4. Center for Thrombosis and Hemostasis (CTH), University Medical Center Mainz, Langenbeckstrasse 1, 55131 Mainz, Germany
5. Department of Otorhinolaryngology, University Medical Center Bonn, Bonn, Germany

* Equal first author contribution, [#]Correspondence: rstauber@uni-mainz.de; ayakhamis@uni-mainz.de. Phone: +49-6131176030.

Supplementary Tables

Table S1. Antibodies used for Western Blot and immunofluorescence analyses.

Antigen	Host	Manufacturer and Article Number	Dilution (x-fold)
**Western Blot Analysis**			
**Actin**	rabbit	Sigma Aldrich, A2066	5000
**E-Cadherin**	mouse	BD Bioscience, 610182	5000
**VDR**	mouse	Santa Cruz; sc-13133	1000
**GAPDH**	mouse	Santa Cruz, sc-47724	3000
**VDR**	rabbit	Abcam, ab3508	5000
**BIM**	rabbit	Sigma, B7929	1000
**⚜0009**	rabbit	Bethyl Laboratories, A300-081A	1000
**Rabbit (HRP coupled)**	goat	Cell Signaling; 7074	5000
**Mouse (HRP coupled)**	mouse	Cell Signaling; 7076	5000

**Immunofluorescence**			
**VDR**	rabbit	Abcam ab3508	200
**Rabbit (FITC coupled)**	goat	Dianova; 111-165-003	300

Table S2. Primer Sequences

Name	Sequence (5‘ → 3‘)
**VDR_fw**	TACCGAGCTCGGATCCCCTGGGCTCCACTTACCTG
**VDR_rev**	CCTCGCCCTTGCTAGCGGAGATCTCATTGCCAAACAC
**ColPCR_pC3_fw**	CCCACTGCTTACTGGCTTAT
**ColPCR_pC3_rev**	AGCAGTACGATCTGGTCCT

Supplementary Figures

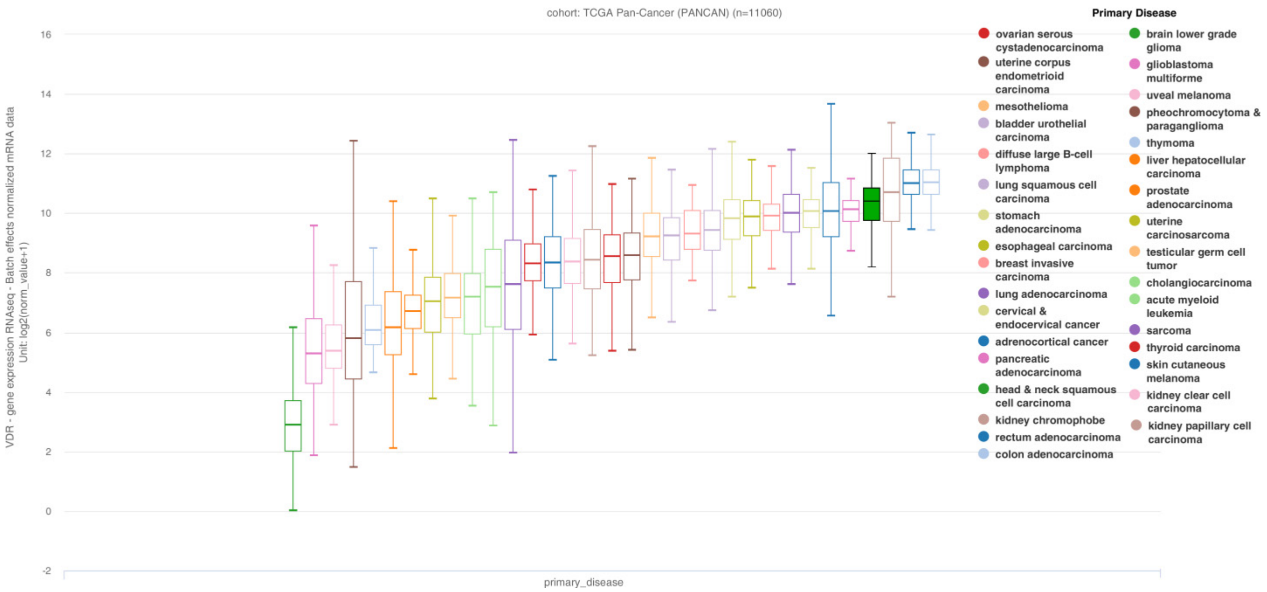

Figure S1. VDR mRNA expression in different tumor types according to the PANCAN data set obtained from *The Cancer Genome Atlas* (TCGA). VDR is highly expressed not only in colon adenocarcinomas, but also in HNSCC, supporting a pathobiological relevance of VDR for this tumor entity.

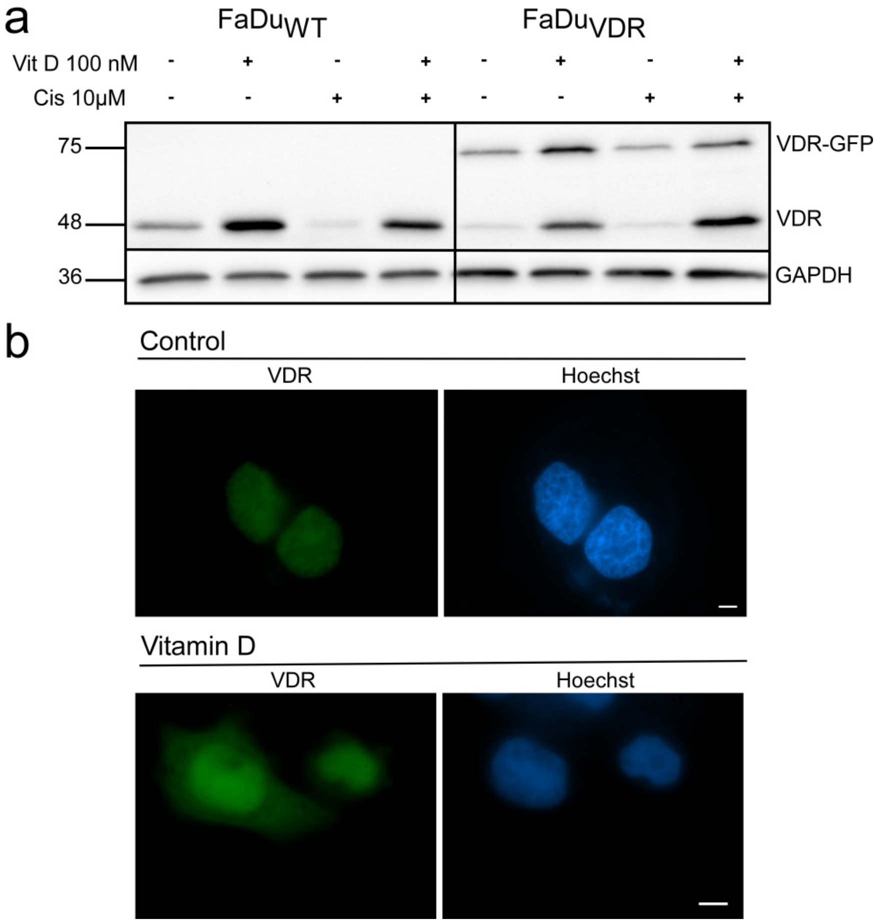

Figure S2. Overexpression of VDR increases cisplatin resistance of FaDu cells. (a) Immunoblot analysis demonstrated successful expression of VDR-GFP in FaDu cells. Furthermore, VDR expression is increased in response to VitD and combination treatment. GAPDH served as the loading control. (b) Fluorescence microscopy to visualize the influence of Vitamin D on VDR receptor expression and translocation in FaDu VDR cells. Nuclei were stained with Hoechst (blue). Scale bar, 5 μm.

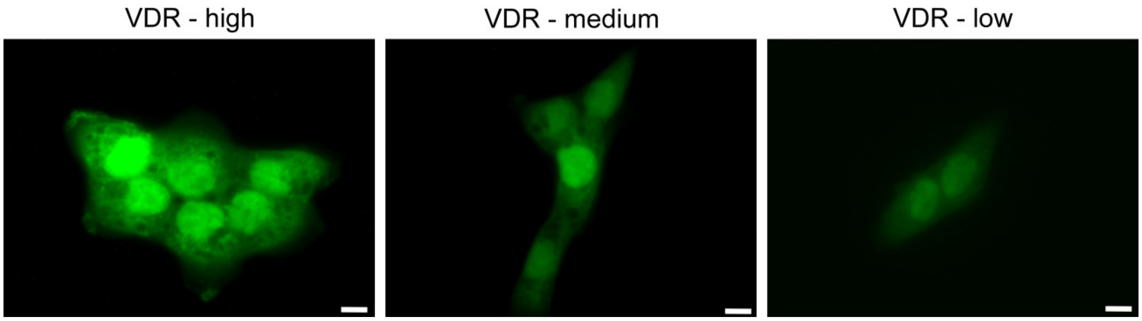

Figure S3. Overexpression of VDR increases cisplatin resistance of HNSCCUM-02T cells. Fluorescence microscopy to visualize the influence of Vitamin D on VDR receptor expression in HNSCCUM-02T- VDR cells. Nuclei were stained with Hoechst (blue). Scale bar, 5 μm.

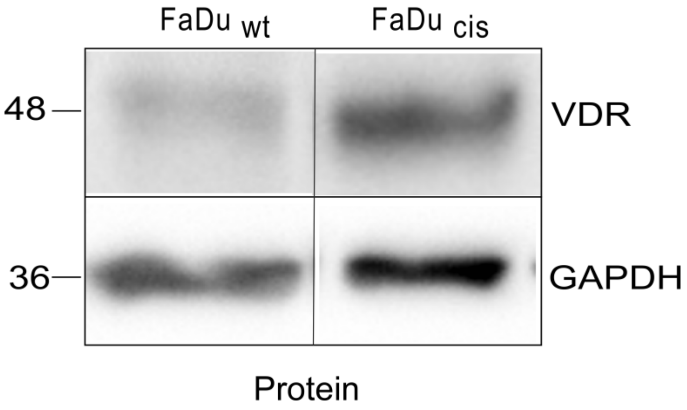

Figure S4. VDR is upregulated in cisplatin-resistant HNSCC cell models. Overexpression of VDR in resistant FaDu $_{CIS}$ versus sensitive (FaDu $_{WT}$) cells was demonstrated by Immunoblot analysis GAPDH served as the loading control.

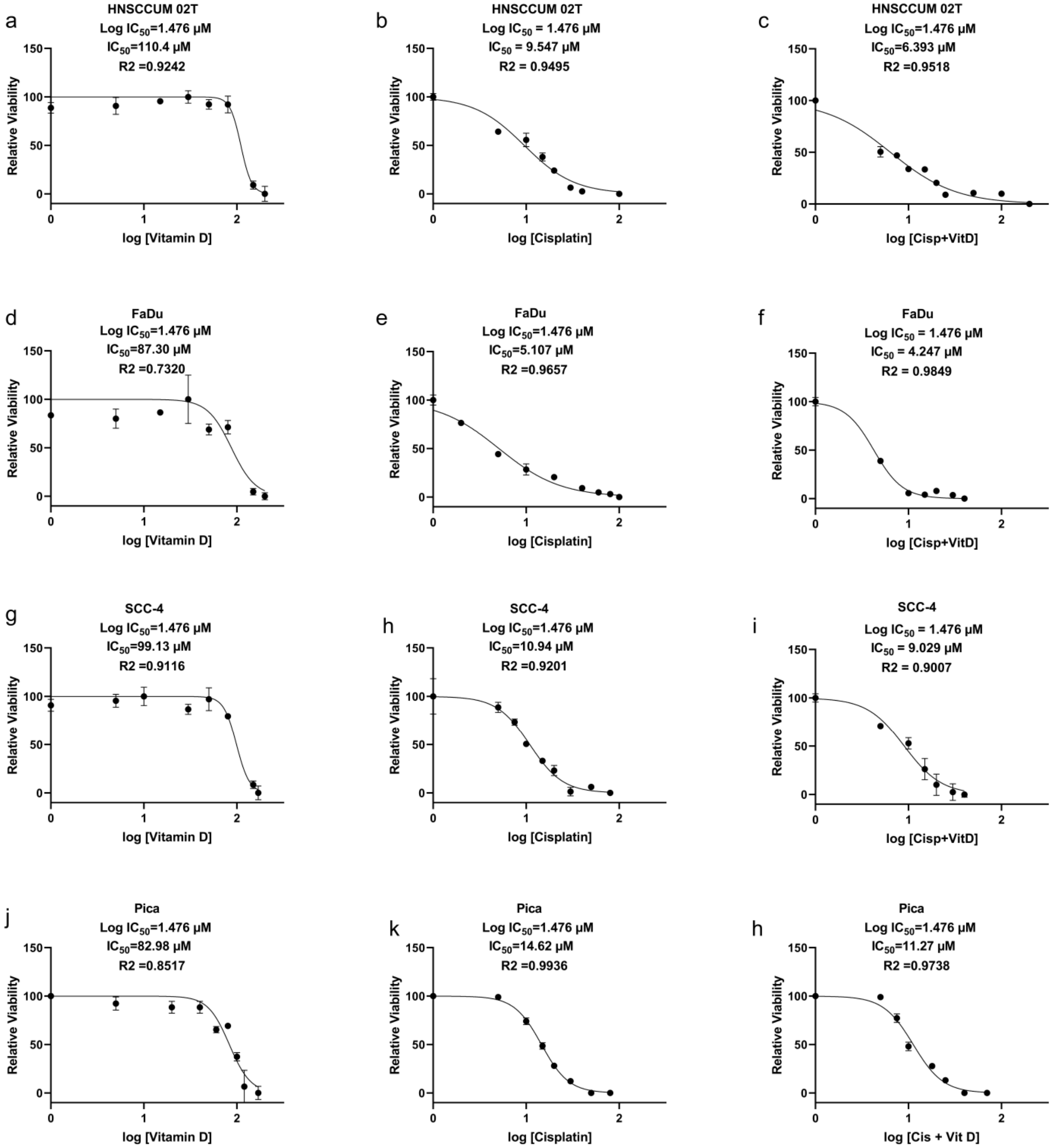

Figure S5. Dose-response curves and IC_{50} values for HNSCCUM-02T (a-c), FaDu (d-f), SCC-4 (g-i), and Pica cells (j-h).

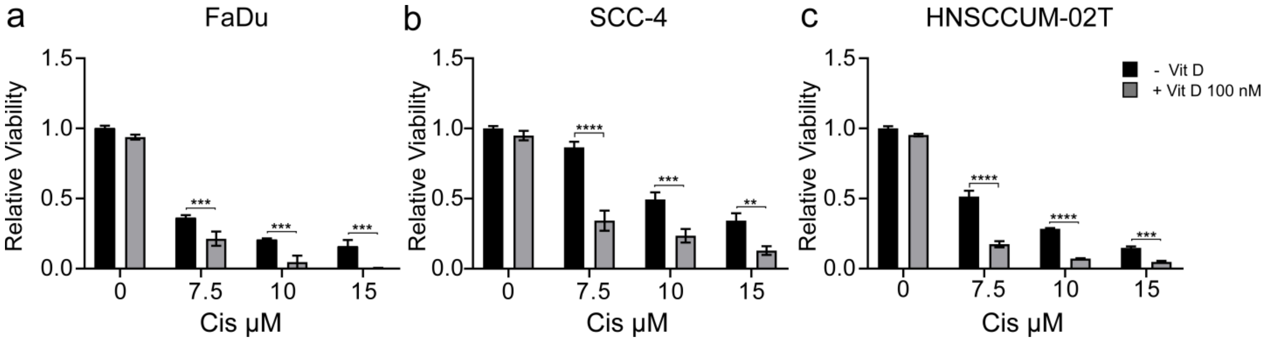

Figure S6. VitD treatment increases cisplatin sensitivity of different HNSCC cell lines. Combinational treatment of VitD/cisplatin synergistically triggered cell death of wild-type (a) FaDu (b) SCC-4 and (c) HNSCCUM-02T cells. Cells were seeded in the presence or absence of 100 nM VitD, after 24h the cells were treated with 7.5, 10, and 15 µM cisplatin with and without VitD. The viability of the cells was measured and normalized to untreated controls.

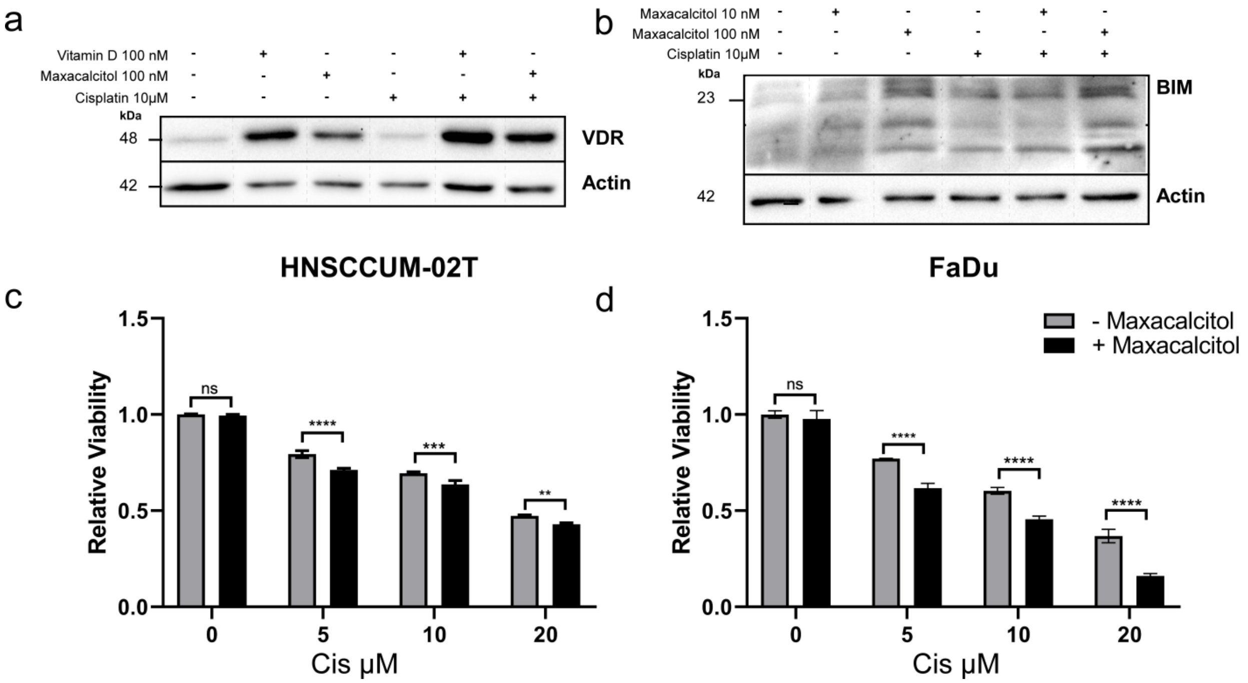

Figure S7. The VitD analog Maxacalcitol executes similar function on HNSCC cells in overcoming cisplatin resistance (a) Immunoblot analyses reveal significant increase of VDR in VitD and Maxacalcitol as well as in Cisplatin Combination co-treated HNSCCUM-02T cells. Actin served as the loading control. (a) Immunoblot analyses reveal significant increase of BIM in Maxacalcitol and Maxacalcitol/Cisplatin co-treated HNSCCUM-02T cells. Actin served as the loading control. (c&d) HNSCCUM-02T and FaDu cells were treated for 72 h (5, 10, and 20µM cisplatin in presence and absence of 100 nM Maxacalcitol). Cell viability was normalized to untreated controls.

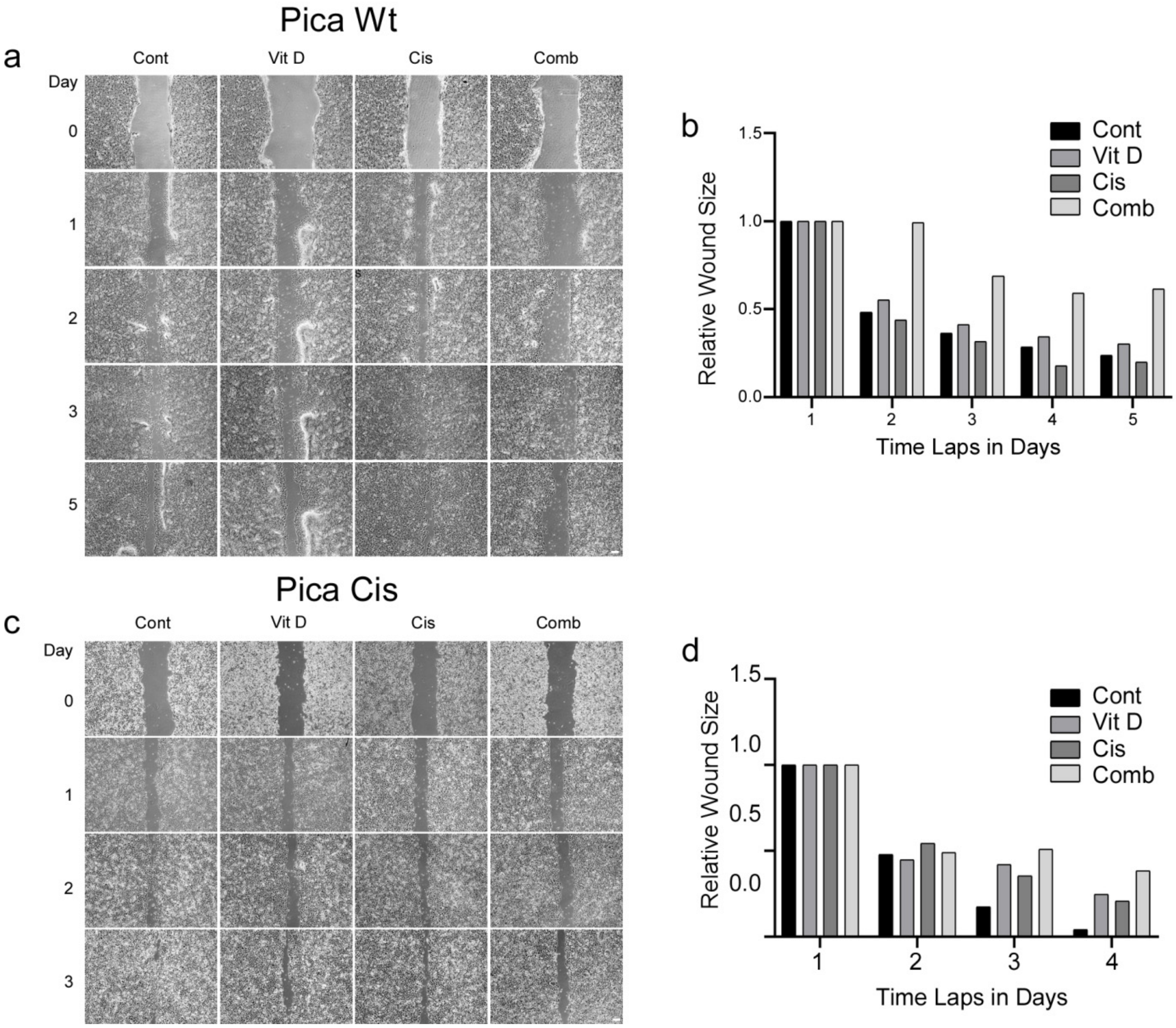

Figure S8. VitD reduces the migration ability of HNSCC cells. (a&c) Wound healing assays were performed to evaluate the migration of Pica Wt, and cisplatin-resistant cells. Cells were treated with VitD (100 nM), or cisplatin (10 μM) alone, and with the combination. Scale bar, 100 μM. (b/d) Relative would size was measured every 24 hrs.

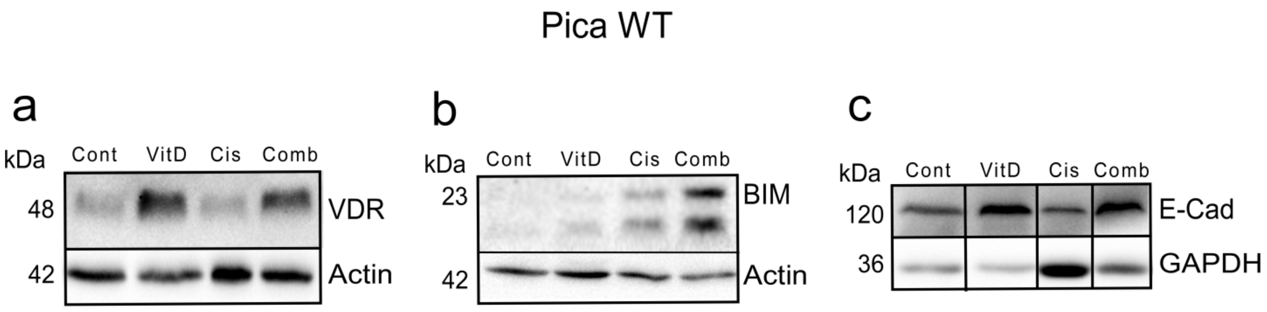

Figure S9. Immunoblot analysis of Pica cells shows upregulation of VDR (a), BIM (b), and E-cadherin (c) in response to VitD/cisplatin. Actin and GAPDH served as the loading control. Proteins were detected by specific Ab.

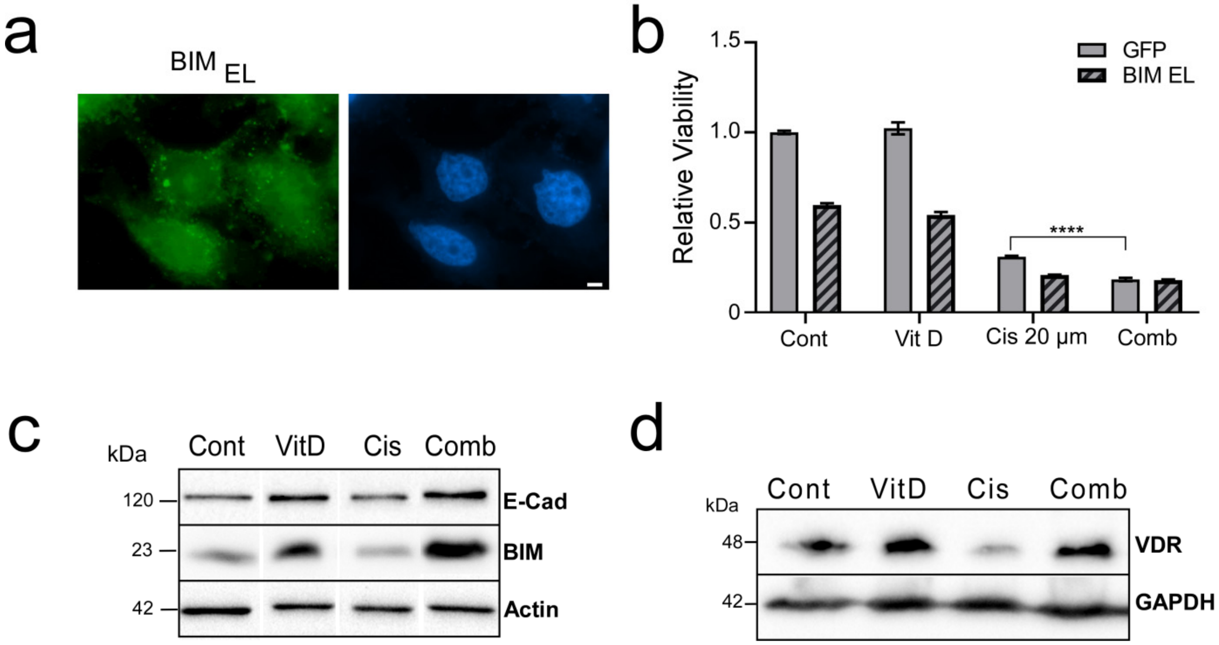

Figure S10. The VitD-BIM axis aids in overcoming cisplatin resistance in Fadu cells. (a) Fluorescence microscopy to visualize cytoplasmic BIM-GFP expression in Fadu cells. Cells were analyzed 16 h *post*-transfection. Scale bar, 10 μm. (b) Ectopic BIM-GFP expression triggers apoptosis of FaDu cells. GFP was used as control. Cells were treated for 72 h (100 μM VitD; 20μM cisplatin, and combination). Cell viability was normalized to untreated controls. (c)&(d) Immunoblot analysis of FaDu cells shows upregulation of E-Cadherin, and BIM (c), and VDR (d) in response to VitD/cisplatin. Actin and GAPDH served as the loading control. Proteins were detected by specific Ab.

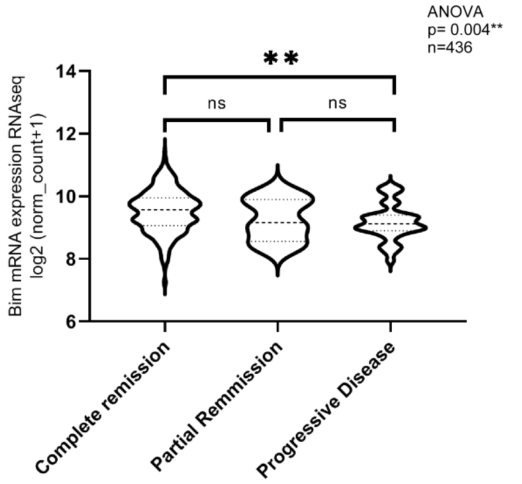

Figure S11. High BIM expression significantly correlates with improved therapy success shown by primary therapy outcomes. P-values and sample size (n) are indicated.
